# Tooth wear in aging people: an investigation of the prevalence and the influential factors of incisal/occlusal tooth wear in northwest China

**DOI:** 10.1186/1472-6831-14-65

**Published:** 2014-06-05

**Authors:** Bo Liu, Min Zhang, Yongjin Chen, Yueling Yao

**Affiliations:** 1 Department of Oral Medical Center, The 174th Hospital of PLA, No.94 Wen Yuan Road, Xiamen, China; 2 Department of General Dentistry and Emergency, College of Stomatology, The Fourth Military Medical University, No.145 ChangLe West Road, Xi'an, China; 3 State Key Laboratory of Military Stomatology, Department of Prosthodontics, School of Stomatology, Fourth Military Medical University, No.145 ChangLe West Road, Xi'an, China

**Keywords:** Tooth wear, Attrition, Erosion, Abrasion, Abfraction

## Abstract

**Background:**

The aim of this study was to estimate the prevalence of tooth wear in the aging population of northwest China and to investigate the factors associated with such tooth wear.

**Methods:**

Cross-sectional analytic clinical and questionnaire study was performed in 704 participants who had a mean age of 46.5 ± 0.2 SD and of which 367(52.13%) were males and 337(47.87%) female. These participants were invited when they attended the hospital which located in northwest China for routine oral examination.

**Results:**

In the maxilla of the examined patients, the rate of tooth wear varied from 85.51% for molar group, 89.77% for premolar group, 100.0% for canine group to 87.22% for incisor group. In the mandible, the rates were 86.36%, 88.92%, 100.0% and 91.19% for the four groups respectively. Moreover, both the incisor and canine groups of these patients showed median scores of 3, the premolar group showed a median score of 1, and the molar group had a median score of 2. Additionally, multiple factors were considered to contribute to these patterns of tooth wear, especially the habitual consumption of a hard or sour diet (P < 0.05,odds ratio 1.21, 95% confidence intervals 1.04-1.49).

**Conclusions:**

Tooth wear is a common disease in which the anterior teeth exhibit greater wear than posterior teeth. The data support an association between tooth wear and dietary patterns.

## Background

The incidence of natural tooth retention is increasing, and consequently, a greater prevalence of tooth wear is observed in the aging population [1]. The term ‘tooth wear’ is used to describe the loss of hard tooth tissue caused by friction between the occlusal surfaces of opposing teeth or between a tooth’s occlusal surface and food during masticatory and non-masticatory movements, with no occurrence of dental caries or trauma. There are three main mechanisms of tooth wear, namely, erosion, attrition, and abrasion [2]. Attrition is the physiological wearing of dental hard tissues through tooth-to-tooth contact, without the intervention of foreign substances [3]. Abrasion is the pathological wear of dental hard tissue through abnormal mechanical processes that involve foreign objects or substances that are repeatedly introduced to the mouth and contact the teeth. Erosion is the loss of dental hard tissues by the chemical dissolution of enamel or dentin through the action of nonbacterial acid from dietary or gastric sources.

A review of the literature reveals that many different tooth wear indices have been developed for clinical and laboratory use all over the world. The literature abounds with many different methods, which can be broadly divided into categories that are quantitative and qualitative in nature [4]. Quantitative methods tend to rely on objective physical measurements, such as depth of grooves, area of facets, and height of crowns [4]. Qualitative methods, which rely on clinical descriptions, can be more subjective and incline to descriptive assessment measures, such as mild, moderate or severe [4]. In this paper, the Tooth Wear Index (TWI), introduced by Smith and Knight was used [5].

Currently, tooth wear is perceived internationally as an ever-increasing problem, especially in the elderly, as it is more common in this age group [6]. Furthermore, dietary habits, the presence of acid reflux and socio-economic status have all been shown to affect the prevalence of tooth wear. However, there are not many studies in China that clearly establish the prevalence and etiology of tooth wear. This study first proposed the types and the distribution of tooth position and tooth wear in Chinese people, especially among the northwestern region of China. The objective of this study was to investigate the prevalence of tooth wear and to assess the possible influential factors associated with such wear among the aging population. This information will enable professionals and public health personnel to establish methods and develop preventive strategies for the passive management of tooth wear in this age group.

## Methods

### Patients

Seven hundred and fifty patients attending the stomatological hospital of the Fourth Military Medical University(FMMU, Shaanxi Xi’an) for routine oral examination between April 2012 and June 2013 were invited to take part in the study. Forty six patients declined participation in the investigation. Therefore, seven hundred and four patients (52.13% males and 47.87% female) with tooth wear, aged 40–50 years (mean 46.5 ± 0.2 SD), were included in this study. In each patient, less than two teeth were missing in either the maxilla or the mandible, and the occlusion of the remaining tooth was normal. Clinical oral examination of the patients was performed in an outpatient dental clinic using a disposable dental mirror, disposable explorer and gauzes (to remove food debris, if necessary), under standard illumination from a dental operating light. All patients were examined intraorally by the same practitioner. In our study, the examiner was trained and calibrated both on clinical intra-oral photograhs and on a group of patients. The intra-examined variations were evaluated according to the World Health Organization (WHO) recommendation giving a Kappa agreement at the end of the training phase of 0.75 as described by Bartlett DW et al. [7]. The authors declare that the protocols were approved by the Human Experimental Ethical Inspection of FMMU (No. IRB-REV-2011015), and that the study was performed in accordance with the Declaration of Helsinki (2008) for humans.

### Clinical examination

The patients gave their informed consent for the use of their data and were willing to answer a questionnaire. The incisal/occlusal surfaces of all teeth were scored according to the criteria shown in Table 1, which were based on the TWI of Smith and Knight as described by Lopez-Frias FJ [4]. This TWI is a comprehensive system in which all four visible surfaces (buccal, cervical, lingual and occlusal/incisal) of all teeth present are scored for wear. The third molar and restored or carious teeth (1284, 7.2%) were excluded from the analysis. The total number of examined teeth was 17712, and all teeth were divided into four groups: the incisor, canine, premolar and molar groups. The incisor group included the central and lateral incisors of the maxilla and mandible; the canine group included the canines of the maxilla and mandible; the premolar group included the first and second premolars of the maxilla and mandible; and the molar group included the first and second molars of the maxilla and mandible. Scores of 0–4 were assigned to the teeth, according to the severity of wear.

**Table 1:** Smith and Knight tooth wear index: B = buccal; L = lingual; O = occlusal; I: incisal

**Score**	**Surface**	**Criteria**
0	B/L/O/I	No loss of enamel surface characteristics
1	B/L/O/I	Loss of enamel surface characteristics
2	B/L/O	Loss of enamel, exposing dentin on less than one-third of surface
	I	Loss of enamel, just exposing dentin
3	B/L/O	Loss of enamel, exposing dentin on more than one third of surface
	I	Loss of enamel and substantial loss of dentin
4	B/L/O	Complete enamel loss, pulp exposure, secondary dentin exposure
	I	Pulp exposure or exposure of secondary dentin

### Questionnaire

Following the clinical examination, a self-administered questionnaire was completed, which was designed based on procedures found in the literature and expert opinion. Information was elicited regarding the presence of bruxism, the consumption of hard or acidic foods, parafunctional activity, working environment (related to dust or acid gas), clicking of the temporomandibular joint, stiffness or fatigue of the masticatory muscles, and acid reflux. Examples of these questions are shown in Table 2. In order to complete the questionnaire, the room mates or family members of the patients were asked to help with the questionnares involved in bruxism, the consumption of hard or acidic foods and others. Most questions required a ‘mostly’, ‘sometimes’ or ‘never’ response.

**Table 2:** Questionnaire responses among 704 patients (%)

	**Mostly**	**Sometimes**	**Never**
Q1: Do you often make tooth grinding sounds during sleep (confirmed by room mate or family member)?	40.91	0	59.09
Q2: Do you favor the consumption of hard or acidic foods?	30.68	21.59	47.73
Q3: Does your work environment involves dust or acid gas?	0	3.41	96.59
Q4: Do you have parafunctional activity, such as clenching or grinding your teeth?	20.45	0	79.55
Q5: Do you suffer from clicking of the temporomandibular joint?	36.36	0	63.64
Q6: Do you suffer from stiffness or fatigue of the masticatory muscles?	9.09	0	90.91
Q7: Do you suffer from acid reflux?	0	4.55	95.45

### Statistical method

The analysis of data was carried out using the Statistical Package for the Social Sciences (SPSS, Inc. Chicago, IL, USA version 10). The relationship between tooth wear and questionnaire items was evaluated in a multiple logistic regression model,estimating an odds ratio per unit increase of the mean tooth wear score when the mean tooth wear was a linear variable in the statistical model. The odds ratio represented the odds of suspected items for an individual with level y mean tooth wear score + 1 unit mean tooth wear score versus the odds of the items for an individual with level y mean tooth wear score. Thereby, the criterion for the independent variables to enter the model was set at 0.2 and the criterion to stay at 0.25. Statistically significant levels were set at *p* <0.05.

## Results

The prevalence rates of tooth wear in dental patients were calculated. In the maxilla, the rate varied from 85.51% for molar group, 89.77% for premolar group, 100.0% for canine group to 87.22% for incisor group. In the mandible, the rates were 86.36%, 88.92%, 100.0% and 91.19% for the four groups respectively. However, no significant difference was observed among these groups in either maxilla or mandible.

The wear severity of four groups in the maxilla and mandible were also measured (Table 3). In the maxilla, the wear severity between the incisor group and canine group exhibited no significant difference (*p* > 0.05); the wear severity of the maxillary incisor group and of the canine group was greater than that of the molar group, which was, in turn, greater than that of the premolar group (*p* < 0.05). In the mandible, no significant difference of the wear severity (*p* > 0.05) was found between the incisor group and of the canine group; However, the wear severity of mandibular incisor group and of the canine group was greater than that of the molar group, which was, in turn, greater than that of the premolar group (*p* < 0.05).

**Table 3:** The scoring of tooth wear

	**Maxilla**				**Mandible**			
**Groups**	**Molar**	**Premolar**	**Canine**	**Incisor**	**Molar**	**Premolar**	**Canine**	**Incisor**
n (%)	602 (85.51)	632 (89.77)	704 (100.0)	614 (87.22)	608 (86.36)	626 (88.92)	704 (100.0)	642 (91.19)
mean score	2.08 ± 0.73	1.55 ± 0.81	2.44 ± 0.65	2.37 ± 0.76	2.24 ± 0.63	1.61 ± 0.75	2.47 ± 0.72	2.55 ± 0.83
distribution	1-3	1-3	1-4	2-4	1-3	1-3	1-4	1-4

All participants completed the questionnaire. Table 2 demonstrates that for the etiological factors that are specifically associated with tooth wear. Notably, Consuming of hard or acidic foods (52.3%), bruxism during sleep (40.9%), and clicking of the temporomandibular joint (36.4%) turn to be the top 3 factors which may be responsible for tooth wear.

The result of multiple logistic regression analysis shows that the preference for hard or acidic foods was significantly associated with tooth wear (*p* = 0.024 < 0.05). The odds ratio is 1.21, which means that consuming of hard or acidic foods increased the chance of tooth wear by 121% (95% confidence intervals 104-149%).

## Discussion

There is a well-recognized trend for increased longevity amongst the population of China, resulting in an increased proportion of aging and elderly people in the community. Concomitant with the observed increase in the proportion of elderly people, there has been a decrease in the rate of tooth loss with increasing age. These two factors have combined to produce a substantial increase in the numbers of aging and elderly patients with some retained teeth. Tooth surface loss is a macroscopically irreversible process that accumulates with age. Lambrechts estimated the normal vertical loss of enamel from physiological wear to be approximately 20–38 μm per annum [8]. When the process of tooth wear is excessive, it leads to tooth shortening, exposed dentin, tooth hypersensitivity, or, more seriously, exposure of the canal, pulpitis, pulp necrosis, and an unsightly appearance [9,10].

In a recent systematic review of the results of tooth wear by all causes, Van’t Spijker concluded that the percentage of adult patients presenting with severe tooth wear increased from 3% at the age of 20 years to 17% at the age of 70 years, with a tendency to develop more wear with age [11]. Similar results were reported in a large epidemiological study with German dental patients, in which the extent of tooth wear was scored on a scale from 0 to 3; in this study, the mean wear scores increased from 0.6 among 20- to 29-year-olds to 1.4 in 70- to 79-year-olds [12]. The incisal surfaces of canines and incisors, together with the occlusal surfaces of molars and premolars, are the functional surfaces of the dentition. This classification indicates their role in mastication and in providing guidance in excursive movements of the mandible. In this study, incisors and canines showed greater wear than molars, and molars showed greater wear than premolars in both the maxillary or mandibular dentition. The canine and incisor teeth displayed a stronger increase in the severity of wear than did the molar and premolar teeth, with mean wear scores that indicated a loss of enamel and a substantial loss of dentin on the incisal surfaces of these teeth. This result was consistent with the findings of other scholars [13]. The reasons for this higher degree of wear observed in the incisors and canines may include the following: i the enamel of incisors is thinner, and incisors are smaller; ii the active role of incisors and canines in both masticatory and excursive jaw movements during function and parafunction, which may place greater demands upon these teeth than that endured by the larger posterior teeth; iii incisors and canines are, on average, the most frequently retained teeth among older people, which may influence the level of wear to which they are subjected. Between the two arches, the incisal surfaces of the mandibular anterior teeth displayed higher mean wear scores than that of the maxillary anterior teeth, and this result may be attributed to the role of the lower incisal edges during incision and throughout the process of protrusive guidance.

The prevalence of tooth wear varies around the globe, and the etiology of tooth wear is multifactorial [14,15]. In developed countries, the prevalence of tooth wear is on the rise, which could be due to changes in dietary patterns [16,17]. Oral habits are repetitive behaviors in the oral cavity that result in loss of tooth structure, including dietary habits, brushing techniques, bruxism, parafunctional habits and regurgitation. Their effect is dependent on the nature, onset and duration of the habits. The role of acidic foods and drinks is likely important to the progression of tooth wear. There is a considerable body of evidence from laboratory studies that indicates that low pH acidic foods and drinks cause erosion of enamel and dentin [18-20]. The coarseness or grit of the diet during function is a main causative factor in occlusal wear. Bruxism is thought to affect 5-20% of the normal population, and it is associated with tooth wear [21]. Pavone noted that abnormal clenching and grinding habits produced unusual wear patterns of occlusal surfaces, and Christensen showed that people who displayed bruxism could experience up to four times more tooth wear than those without this habit [22,23]. Individuals with stronger and/or more frequent bite forces should exhibit more tooth wear. In this study, the survey results found that eating habits, bruxism and joint disease constitute the majority of the total respondents, followed by parafunctional activity, the presence of reflux disease and working conditions. Analyzing the relevant factors that affect tooth wear by multiple logistic regression analysis, it was found that the preference for hard or acidic food had the greatest effect on tooth wear. The constituents of the diet, the consistent chewing of abrasive diets, the presence of unglazed enamel and environmental factors, such as constant exposure to dust and grit in farming activities, were related to abrasion [24]. Eisenburger found that simultaneous erosion and abrasion resulted in approximately 50% more wear than alternating erosion and abrasion [25]. Among these seven hundred and four patients, one male subject had retained twenty-six teeth, and he had twenty-one teeth with wear scores of 3 to 4. His questionnaire revealed that the frequently consumed Daguokui (known to be dry, hard, and chewy) and Shaanxi sour soup noodles and that his working environment involved dust (miner). This finding may indicate that softened enamel is highly unstable and that it can be easily removed by short and relatively gentle physical action. Therefore, the chewing of acidic foods with a stronger bite force might cause enhanced tooth wear.

## Conclusions

In conclusion, this study has provided data on the proportions of the various types of tooth wear lesions among aging people in northwest China. Though the observed patterns of wear displayed no differences from those encountered in Western cultures, the preference for hard or acidic food turn to be the major cause in northwest China. Knowledge of the etiology of such lesions is important for the prevention of further lesions and the termination of the progression of already-present lesions. According to the epidemiological analysis of tooth wear performed in this study, people of various regions need to take appropriate preventive measurements. This study suggests that it is important to protect and prevent the wear of incisors and canines among people who favor the consumption of hard or acidic foods in the northwestern region of China. In addition, further investigation is required to identify the specific risk factors of tooth wear.

## Abbreviations

TWI: Tooth wear index.

## Competing interests

The authors declare that they have no competing interests.

## Authors’ contributions

M Zhang and YL Yao designed the study. B Liu analyzed the data. B Liu and YJ Chen drafted the paper. All authors read and approved the final manuscript.

## Pre-publication history

The pre-publication history for this paper can be accessed here:

http://www.biomedcentral.com/1472-6831/14/65/prepub
